# Incidence, risk factors and the prognostic role of thromboembolic events (TEEs) amongst patients with metastatic pancreatic adenocarcinoma (PAAD): a retrospective, single-center analysis

**DOI:** 10.3332/ecancer.2024.1738

**Published:** 2024-08-14

**Authors:** Cícero Gonzaga Santos, Francisco de Assis Maia, Marcos Pedro Guedes Camandaroba, Victor Hugo Fonseca de Jesus

**Affiliations:** 1 Department of Medical Oncology, A.C. Camargo Cancer Center, São Paulo, SP 01509-010, Brazil; 2 Department of Medical Oncology, Centro de Pesquisas Oncológicas (CEPON), Florianópolis, SC 88034-000, Brazil; 3 Department of Gastrointestinal Medical Oncology, Oncoclínicas, Florianópolis, 88015-020, Brazil; 4 Post-Graduate School, A.C. Camargo Cancer Center, São Paulo, SP 01509-010, Brazil

**Keywords:** thromboembolic, thrombosis, embolism, metastatic, pancreatic, cancer

## Abstract

**Background:**

Thromboembolic events (TEEs) are frequent among patients with pancreatic adenocarcinoma (PAAD). We set out to estimate the incidence and establish predictive risk factors for TEE and estimate the impact of TEEs on the overall survival (OS) of patients with metastatic PAAD.

**Methods:**

This is a retrospective, single-center study. We included patients with a pathologically confirmed diagnosis of PAAD with distant metastases treated at AC Camargo Cancer Center from 2016 to 2021. We used the competitive risk survival models to estimate the cumulative incidence of TEE. Risk factors for the development of TEEs were evaluated using the competitive risk and logistic regression models. The impact of TEEs on OS was assessed using both landmark and time-dependent covariate Cox survival analyses.

**Results:**

The study population consists of 199 patients. The cumulative incidence of TEEs in 1, 6 and 24 months were 10.1%, 19.3% and 30.2%, respectively. Log_10_(CA 19-9) was the only factor independently associated with increased risk of TEEs in the logistic regression (Odds ratio = 1.03; 95% confidence interval (95%CI), 1.00–1.06; *p* = 0.030) and competitive risk survival (Subdistribution hazard ratio = 1.14; 95%CI, 1.02–1.27; *p* = 0.019) models. In the landmark analysis, early TEEs (within 1 month of diagnosis) were not associated with inferior OS. In the time-dependent covariate Cox proportional hazard model, TEEs were not found to be statistically associated with inferior OS, although there was a trend towards it (Hazard ratio = 1.59; 95%CI, 0.99–2.54; *p* = 0.051).

**Conclusion:**

TEEs occur in a large fraction of patients with metastatic PAAD. Statistical models with higher predictive performance are currently needed. For the time being, consideration for prophylactic anticoagulation should be done on an individual basis.

## Introduction

Pancreatic adenocarcinoma (PAAD) is the 6th most common cause of cancer-related death worldwide [1]. Its epidemiological burden is growing in both developed and developing countries [2, 3], and PAAD is expected to become the 2nd most common cause of cancer-related death in the USA by 2026 [4]. Indeed, among women, PAAD has already surpassed colorectal cancer as the 3rd most common cause of cancer-related death in the USA [5]. Therefore, there is a need for a deep understanding of the mechanism underpinning PAAD morbidity and mortality.

Thromboembolic events (TEEs) are one of the most common clinical complications among patients with PAAD. In contemporary series, TEEs have been shown to occur in 14%–35% of patients with PAAD [6–14], the highest incidence rate amongst all human malignancies [15]. These rates are even higher when we consider patients with metastatic disease [6, 10, 16]. The high frequency of TEEs amongst patients with metastatic PAAD has spurred the use of prophylactic anticoagulation amongst these patients. Indeed, several randomised clinical trials (RCTs) have shown a significant decrease in the incidence of TEEs for patients with solid tumours (including PAAD) undergoing prophylactic anticoagulation [17–21].

However, despite the significant burden of TEEs in patients with PAAD, autopsy series show that TEEs rarely are the underlying mechanism of death of patients with PA [22, 23], and this holds especially in recent years when better imaging techniques have become available to diagnose TEEs [23]. Perhaps, this minor role of TEEs in PAAD-associated mortality explains the disappointing overall survival (OS) outcomes of RCTs assessing the role of prophylactic anticoagulation for patients with metastatic PAAD [17, 18].

Therefore, the net benefit of prophylactic anticoagulation for patients with metastatic PAAD remains disputable. To better understand its potential usefulness, we set out to investigate the frequency and prognostic role of TEEs amongst patients with PAAD treated at the A.C. Camargo Cancer Center and also looked for potential risk factors for the development of TEEs in this population.

## Methods

### Study design

This is a retrospective, unicentric, descriptive and analytic study. We gathered routinely collected data from the electronic medical records of patients with metastatic PAAD. Due to its retrospective nature, the need for an informed consent term was waived. This study was approved by the A.C. Camargo Cancer Center Internal Ethics Committee (CAAE: 65909422.5.0000.5432).

### Population

We included patients aged 18 years old and above with pathologically confirmed diagnosis of PAAD with radiological or pathological confirmation of metastatic disease from 1st January 2016 to 31st December 2021. We excluded patients who received chemotherapy or the best supportive care in other institutions and those with concurrent metastases from other solid tumours.

### Variables

We collected demographic, clinical and pathological data from patients’ electronic medical records. We identified patients who experienced TEEs by searching clinical notes and imaging reports within patients’ electronic medical records. Thereafter, we collected details regarding TEEs (such as type of TEE (arterial versus venous), TEE site and symptoms at TEE diagnosis).

### Procedures

At the diagnosis of metastatic disease, patients were staged using computed tomography (CT) and/or magnetic resonance imaging (MRI). Patients undergoing chemotherapy underwent routine assessment of chemotherapy activity with CT and/or MRI every 8–12 weeks. They were not screened for TEEs using other imaging modalities (such as color Doppler ultrasound) unless they were symptomatic.

### Outcomes

The study’s primary outcome was the incidence of TEEs. TEEs were defined as any radiologically proven thrombus affecting the arterial, venous, or intracardiac compartments, with or without symptoms (incidental). We included TEEs that occurred one month before the diagnosis of metastatic PAAD and thereafter. OS was defined as the time from the diagnosis of metastatic disease to death or last follow-up visit. We considered that the TEE was the likely cause of death when this was the primary diagnostic hypothesis leading to the patient’s demise according to the treating physician’s impressions or review of the patient’s medical record [24]. Significant bleeding during anticoagulation was defined as a drop of 2 or more hemoglobin points secondary to bleeding [25]. A recurrent TEE was defined as a thrombosis and/or embolism affecting a previously uninvolved site or one that had (clinical or radiological) interval documentation of resolution [26].

### Statistical analysis

We used absolute values and ratios to describe the distributions of categorical variables and median values and interquartile ranges (IQRs) to outline the distribution of numerical variables. We employed the competitive risk survival model (risk of TEE versus risk of death) to describe the cumulative incidence rates of TEEs [27]. Survival curves were generated using the Kaplan-Meier method and compared using the log-rank test. Median follow-up was calculated with the reverse Kaplan-Meier method. Patients without an event were censored at the last visit. Patients whose TEE was diagnosed up to a month before the detection of metastatic disease had the date of TEE set to the date of diagnosis of metastases. We generated univariate and multivariate logistic regression (with odds ratios (ORs)) and competing risk survival (with subdistribution hazard ratios (SHRs)) models to look for predictors of TEEs. We evaluated the performance of baseline cancer antigen 19-9 (CA 19-9) levels in predicting TEE using the receiver operating characteristic (ROC) curve (calculated through the closest topleft model with 95% confidence intervals generated with 2,000 bootstrap replicates). We assessed the prognostic role of early TEE (≤ 1 month from diagnosis of metastatic disease) in OS in a landmark analysis. We excluded patients who died in the first month after the diagnosis of metastatic disease and assessed the prognostic role of TEEs within the first month of diagnosis. Also, as TEE status can vary during patients’ disease course (from no TEE in metastatic disease to TEE during metastatic disease), we employed time-dependent covariate Cox proportional hazard models to look for the prognostic role of TEE. We assumed that Eastern Cooperative Oncology Group (ECOG) performance status, presence of liver metastasis, CA 19-9 levels, number of metastatic sites and TEE were time-dependent covariates. Here, we excluded patients with missing values of CA 19-9 (a constraint of the survival package for the time-dependent covariate Cox proportional hazard model). Variables with *p*-values <0.25 in the univariate analyses were used to build multivariate models [28]. A two-tailed *p*-value <0.05 was considered statistically significant. Statistical analyses were performed using the software project R version 4.3.2 (packages *tidycmprsk* and *survival*).

## Results

### Study population

**Supplementary Figure 1.**
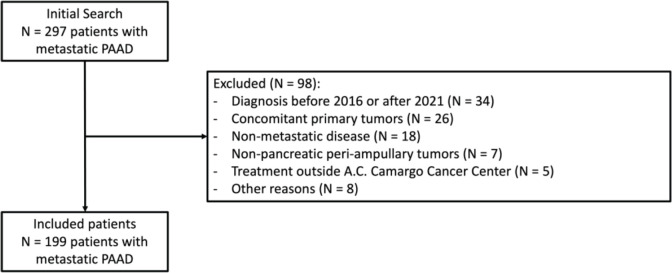
Flow diagram of study population.

We identified 297 patients with metastatic PAAD in our database search. We excluded 98 patients for the following reasons: diagnosis of metastatic disease before 1st January 2016 or after 31st December 2021 (34 patients), concomitant primary tumours (26 patients), nom-metastatic disease (18 patients), diagnosis of non-pancreatic peri-ampullary tumour (7 patients), treatment outside A.C. Camargo Cancer Center (5 patients) and other reasons (8 patients). Therefore, the study population consists of 199 patients – Supplementary Figure 1.

Table 1 describes the characteristics of the study population. Median age was 67 years (IQR: 58–73) and males and females were equally represented. Most patients had an ECOG performance status of 0 or 1 (*N* = 168; 84.5%) at the diagnosis of metastatic disease. Most patients presented with *de novo* metastases (*N* = 131; 65.8%) and liver metastases were present in 64.8% (*N* = 129) of the patients. The median CA 19-9 level was 540.0 UI/mL (IQR: 74.8–4995.8). Thirty-seven patients (18.6%) had a personal history of TEE and 55 patients (27.7%) were on anti-platelet drugs and/or anticoagulants at baseline. Most patients (*N* = 121; 60.8%) were classified as intermediate risk according to the Khorana risk score.

### Characteristics of the TEEs

**Figure 1.**
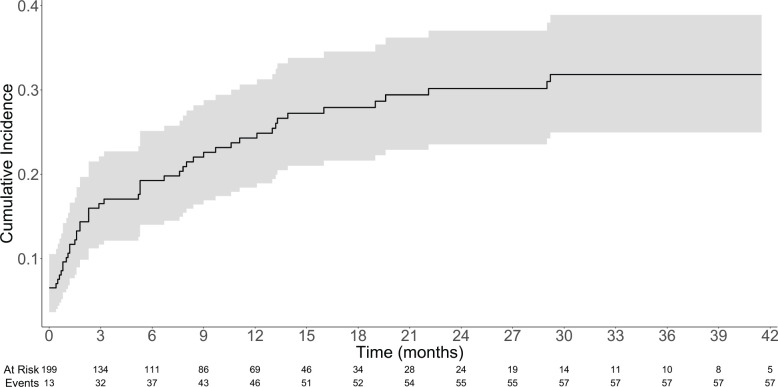
Cumulative incidence of TEEs with 95% confidence interval.

Overall, 58 patients (29.1%) developed TEEs. The median time from diagnosis of metastatic disease to TTE was 2.3 months (IQR: 0.53–9.53). The cumulative incidence rates of TEE in 1, 3, 6 and 12 months were 10.1%, 16.5%, 19.3% and 24.3%, respectively – Table 2 and Figure 1. Table 3 describes the characteristics of the TEEs. Pulmonary embolism (PE; *N* = 21; 36.2%) and lower extremity venous thrombosis (LEVT; *N* = 17; 29.3%) were the most frequent types of events. Most PE events (*N* = 19; 90.5%) were central or segmental and most LEVT events (*N*

**Supplementary Figure 2.**
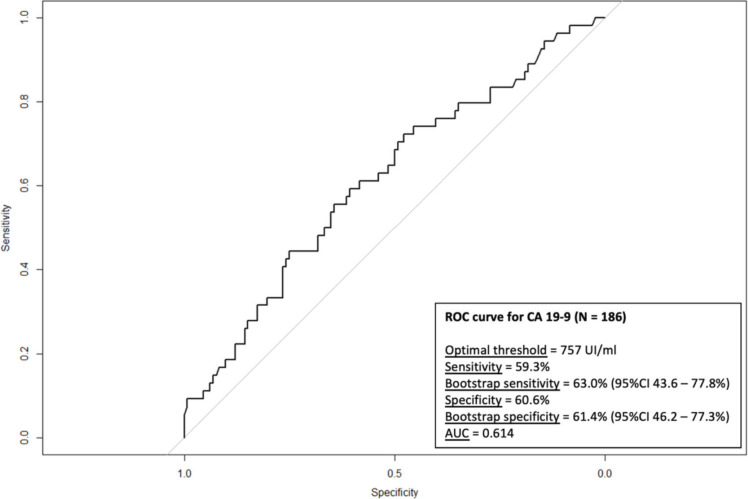
ROC curve for the risk of TEE according to CA 19-9 levels.

= 10; 58.8%) affected deep veins of the lower extremities. Most patients were asymptomatic at the diagnosis of TEEs (*N* = 36; 62.1%). Most TEE episodes occurred before (*N* = 17; 29.3%) or during (*N* = 20; 34.5%) first-line therapy – Supplementary Table 1. When TEEs occurred during chemotherapy, they most often occurred when patients were receiving FOLFIRINOX (*N* = 11; 19.0%) or gemcitabine plus nab-paclitaxel (*N* = 10; 17.2%) – Supplementary Table 2. In both the logistic regression (OR = 1.03; 95% confidence interval (95%CI), 1.00–1.06; *p* = 0.030) and competitive risk survival (SHR = 1.14; 95%CI, 1.02–1.27; *p* = 0.019) models, elevated log_10_(CA 19-9) levels were the only predictor of development of TEEs in the multivariate analyses – Supplementary Tables 3 and 4. Also, in the competitive risk survival analysis, multiple primary sites showed a trend toward higher risk of TEEs (SHR = 2.36; 95%CI, 0.93–5.97; *p* = 0.071). In logistic regression, CA 19-9 levels ≥757 UI/mL were associated with increased risk of TEE (AUC = 0.614; sensitivity = 0.593; specificity = 0.606) – Supplementary Figure 2.

### Outcomes of TEEs

Table 4 describes the clinical outcomes of the 58 patients diagnosed with TEEs. Most patients were managed in the outpatient setting (*N* = 23; 39.7%) or were admitted to the medical ward with no significant clinical repercussions from the TEE (*N* = 21; 36.2%). Three patients (5.2% of patients with TEEs and 1.5% of all patients) had TEEs as the likely cause of death. The most commonly used drugs for the management of the TEE were rivaroxaban (*N* = 27; 46.6%) and enoxaparin (*N* = 16; N = 27.6%). Two patients (3.4%) experienced significant bleeding after anticoagulation. New TEEs occurred in ten patients (17.2%), mostly after withdrawal of anticoagulation (*N* = 6; 10.3%).

### Survival analysis

**Supplementary Figure 3.**
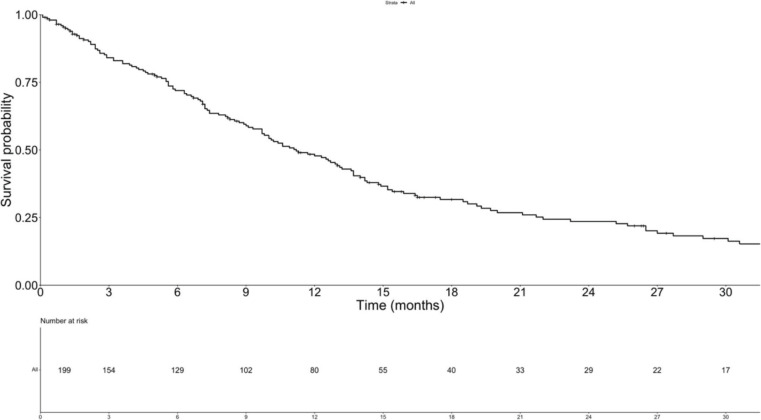
OS of the study population.

Median follow-up was 29.5 months (95%CI, 25.9–40.5). Forty-two patients (21.1%) were lost to follow-up. Median OS was 11.2 months (95%CI, 9.7–13.6)–Supplementary Figure 3. Median OS after the diagnosis of TEE was 3.9 months (95%CI, 1.3–8.5), ranging from 0.3 months in BSC to 9.1 months before first-line–Supplementary Table 5. There was no statistically significant difference between the OS times of patients with asymptomatic (5.6 months; 95%CI, 2.5–10.6) and symptomatic TEEs (1.9 months; 95%CI, 0.5–8.5), although there was a trend toward increased OS in the former group (*p* = 0.07). In a landmark survival analysis, liver metastases (HR = 1.67; 95%CI, 1.11–2.51; *p* = 0.014) and increased log_10_(CA 19-9) levels (HR = 1.09; 95%CI, 1.02–1.17; *p* = 0.013) were associated with worse OS, while TEE in the first month was not – Supplementary Table 6. Of note, among patients who died in the first month after the diagnosis of metastatic disease, five (31.2%) experienced TEEs before death.

In time-dependent covariate Cox proportional hazard models, 35 patients were excluded due to missing values for the CA 19-9 either at diagnosis of metastatic disease or TEE. In the multivariate model, ECOG 2-4 (HR = 1.92; 95%CI, 1.19–3.10; *p* = 0.007), presence of liver metastasis (HR = 1.85; 95%CI, 1.21–2.83; *p* = 0.004) and increased log_10_(CA 19-9) levels (HR = 1.10; 95%CI, 1.02–1.18; *p* = 0.009) were associated with worse OS. TEE was not associated with OS in this analysis despite the *p*-value being close to the statistical significance threshold – Table 5.

## Discussion

In this study, we show that nearly one in three patients with metastatic PAAD will develop TEEs either at diagnosis or thereafter. Most TEEs will be asymptomatic and despite the high incidence of TEEs, they were rarely considered to be the cause of death in our cohort. In our time-dependent covariate Cox proportional hazard model, TEEs were associated with a trend toward inferior OS (not statistically significant). Importantly, the Khorana score was not able to identify patients with metastatic PAAD at the highest risk of developing TEE and only CA 19-9 levels were associated with increased risk of TEEs.

PAAD is associated with the highest rates of TEEs among all malignancies [15]. TEEs have been shown to occur in up to 57%–67% of patients with PAAD in early autopsy series [29, 30] and 14%–35% in contemporary clinical series [6–14]. These variable rates are secondary to differences in patient population (Asian patients experience TEE less often than Western ones) [31], definition of TEE (inclusion or not of arterial thrombotic events), treatment setting (chemotherapy is associated with increased risk of TEE) [9], duration of follow-up and composition of the population. Patients with metastatic PAAD have a 2.5–3.7 times higher risk of developing TEE [8, 16]. Therefore, we believe the relatively high incidence of TEEs found in this study can be explained by the inclusion of patients exclusively with metastatic disease, the use of a broad definition of TEE and the longer follow-up of patients from a Western population.

Given the high frequency of TEEs in patients with advanced PAAD, multiple studies have been undertaken to assess the role of prophylactic anticoagulation in this population. In the FRAGEM and the CONKO-004 studies, in addition to chemotherapy, patients with advanced PAAD

were given prophylactic dalteparin or enoxaparin, respectively [17, 18]. Both studies meet the primary endpoint of reduction in the incidence of TEEs. However, both failed to show improvements in OS for patients treated with prophylactic anticoagulation. While these studies were likely underpowered to evaluate differences in survival outcomes, other factors might explain the lack of OS benefit in favour of prophylactic anticoagulation. Patients with metastatic PAAD undergoing chemotherapy have their disease status checked every 8–12 weeks using CT or MRI. Recent studies suggest 50%–77% of all TEEs occurring in patients with PAAD are incidental [7, 9, 10, 14, 16, 32, 33], and CT and MRI are often able to identify such TEEs. Therefore, patients can be promptly started on therapeutic anticoagulants before they become symptomatic.

In our study, 62% of patients were asymptomatic at the onset of the TEE and these patients had a trend toward longer survival after TEE when compared to those with symptomatic TEE. While there is controversy as to whether incidental TEEs are associated with improved OS when compared to symptomatic episodes, treatment of TEE in the asymptomatic phase theoretically leads to a reduction in the number of deaths directly related to TEE. This might explain data from retrospective studies suggesting the risk of death as a direct consequence of a TEE in patients with PAAD is very low (0%–1.3%) [7, 9, 14, 33]. Accordingly, in the pre-specified analysis of patients with PAAD with high-risk Khorana scores from the CASSINI study, the risk of VTE-related death was only 0.7% in the up-to-date 180 observation period [21]. Our study shows similar findings, with 1.5% of patients dying as a direct consequence of TEE. Therefore, the risk of TEE-related death seems to be rather low in the contemporary management of patients with metastatic PAAD.

In our study, the risk of significant bleeding during therapeutic anticoagulation for patients with TEEs was relatively low at 3.4%. Nonetheless, other retrospective series suggest the risk of bleeding in patients with PAAD undergoing anticoagulation has been underappreciated and that rates of bleeding in clinical trials might underestimate the true incidence of this complication in this population [9, 10]. Indeed, in a large prospective series from Japan, PAAD patients had the highest risk of bleeding among six different tumour types, suggesting these patients might be more prone to bleeding complications [31].

Given that most patients with metastatic PAAD will not develop TEEs, that many of these episodes are asymptomatic, and that these patients might be at greater risk of bleeding, the use of prophylactic anticoagulation could be guided by risk algorithms. The Khorana score has been devised and validated in large cohorts of patients with cancer [34]. In brief, information on primary tumour site, body mass index (BMI), pre-chemotherapy platelet and leukocyte counts and hemoglobin level (or use of red blood cell growth factors) are used to assign patients to a three-tier risk classifier. By definition, all patients with PAAD are classified as having an intermediate or high risk of TEE. However, despite the wide acceptance of the Khorana score, multiple studies have failed to show that the Khorana score adequately predicts the risk of TEEs amongst patients with PAAD [13, 14, 16, 32]. Likewise, we failed to identify the Khorana score as a predictor of the risk of TEE amongst patients with metastatic PAAD. It has been suggested that in the presence of PAAD, many items of the Khorana score have poor predictive value [16]. Moreover, PAAD patients comprised <2% of patients in the original cohort used to develop the score and in the validation cohorts. Finally, one limitation of the Khorana score is that it has been devised to predict the risk of symptomatic TEEs. Indeed, limited data suggest the Khorana score might be accurate only in predicting symptomatic TEEs [13, 35]. Therefore, one cannot recommend the use of the Khorana score to predict the risk of TEEs in patients with metastatic PAAD.

Besides the presence of metastatic disease, multiple risk factors for the development of TEEs amongst patients with PAAD have been suggested. Treatment with chemotherapy, liver metastases [33, 36], previous TEE [31], previous anti-thrombotic treatment [37], obesity (BMI ≥30 kg/m^2^) [31, 37], non-O ABO blood type [37] and primary tumour site in body/tail [7, 16, 37] have all been associated with increased risk of TEE. In our study, the only variable independently associated with increased risk of TEE in both logistic regression and the competing risk survival models was higher baseline CA 19-9 level. Jeong *et al* [7] found among patients with advanced PAAD, those with CA 19-9 levels >1,000 UI/mL at baseline had a 2.7 times higher risk of TEE when compared with those with lower values. Given this cut-off is rather arbitrary, we set out to establish an optimal threshold value based on our logistic regression model. Disappointingly, in our study the performance of baseline CA 19-9 level as an isolated predictor of TEE was modest, suggesting other risk factors and/or the temporal evolution of this tumour marker should be taken into account. In the latter sense, Peippo *et al* [38] suggested that CA 19-9 doubling time could be used to identify those at greater risk of developing TEEs, as patients with TEE show shorter CA 19-9 doubling time than those who do not experience TEE (median: 2.7 versus 16.1 months; *p* = 0.026). One should highlight that while some studies have failed to identify elevated baseline CA 19-9 levels as risk factors for TEEs (perhaps due to issues related to study design) [16, 37], CA 19-9 is associated with thrombin generation in treatment-naïve patients [39] and is an important prognostic factor for patients with metastatic PAAD [40, 41]. Therefore, it seems reasonable to consider CA 19-9 levels in the decision-making process of whether or not to offer prophylactic anticoagulation to patients with metastatic PAAD.

Whether or not TEEs are associated with inferior OS amongst patients with advanced PAAD has been a matter of debate. In a recent meta-analysis, TEEs have been associated with inferior OS (HR = 1.38; *p* < 0.001) and disease-free survival (HR = 2.42; *p* < 0.001) [42]. Interestingly, this worse prognosis seems to be driven by the stronger prognostic impact of early (before therapy initiation) TEEs. In our study, we analysed the prognostic role of TEE in OS using a time-dependent covariate model to account for the fact that TEEs can occur at any time during the clinical evolution of patients with metastatic PAAD. We show that patients with TEEs had a trend toward worse OS (HR = 1.59; *p* = 0.051). Interestingly, in our landmark analysis excluding patients with early death (within 1 month of the diagnosis of metastatic PAAD), early TEEs were not associated with inferior OS, perhaps due to insufficient statistical power or because many of these patients were able to receive active chemotherapy regimens that changed the natural history of the disease. Alternatively, by excluding patients who died within 30 days of the diagnosis of metastatic disease, we might have selected patients with TEE with favourable characteristics, blurring the difference between the two treatment groups. In any case, our study ratifies the negative prognostic impact of TEEs in the disease course of patients with metastatic PAAD.

Our study has limitations. It is susceptible to biases related to its retrospective design and, as a unicentric investigation, we acknowledge our findings might not be immediately applicable to other populations. Also, the relatively modest sample size of this study possibly rendered it underpowered to establish the relationship between some predictive factors and the risk of TEE. Also, we were not able to find a clinically optimal CA 19-9 threshold that identifies patients with the highest risk of developing TEEs. Finally, as patients were not systematically submitted to an autopsy, we might have underestimated the true frequency of deaths secondary to TEE. However, our study has strengths. In our analysis, we included only patients with metastatic PAAD. Moreover, we described in detail the risk and natural history of patients experiencing TEEs. Finally, using statistically sound methods, we confirmed the prognostic role of TEEs in the clinical course of patients with metastatic PAAD.

## Conclusion

Currently, international guidelines suggest prophylactic anticoagulation should be considered for patients at intermediate or high risk of TEEs [43, 44]. However, the slow acceptance of this recommendation translates into the reluctance from many clinicians to administer potentially harmful drugs to patients when RCTs have failed to show any OS benefit. Looking ahead, we should be able to design more accurate prediction models to determine the risk of TEE amongst patients with metastatic PAAD, perhaps using dynamic and molecular biomarkers. This way, we can optimize benefits while minimizing risks, discomfort and costs of prophylactic anticoagulation in this patient population. Until then, we believe a patient-centered discussion using the currently available risk factors for the development of TEEs and bleeding can be used to decide whether the benefits of prophylactic anticoagulation outweigh its potential harms for a given patient.

## List of abbreviations

AUC, Area under the curve; BMI, Body mass index; CA 19-9, Cancer antigen 19-9; CT, Computed tomography; ECOG, Eastern Cooperative Oncology Group; HR, Hazard ratio; ICU, Intensive care unit; IQR, Interquartile range; LEVT, Lower extremity venous thrombosis; MRI, Magnetic resonance imaging; OR, Odds ratio; OS, Overall survival; PAAD, Pancreatic adenocarcinoma; PE, Pulmonary embolism; RCT, Randomised controlled trials; ROC, Receiver operating characteristic; SHR, Subdistribution hazard ratio; TEE, Thromboembolic event; VT, Venous thrombosis.

## Conflicts of interest

The authors have no conflict of interest to declare.

## Funding

This study had no funding source.

## Author contributions

Cícero Gonzaga Santos: conceptualisation, data collection, writing and visualisation; Francisco de Assis Maia Jr: data collection, writing and visualisation; Marcos Pedro Guedes Camandaroba: conceptualisation, writing and visualisation; Victor Hugo Fonseca de Jesus: conceptualisation, statistical analysis, writing and visualisation.

## Figures and Tables

**Table 1.** Overall characteristics of the population.

Variable	*N* = 199 (%)
Age Median (IQR)	67 (58–73)
Sex Male Female	100 (50.3) 99 (49.7)
ECOG performance status ECOG 0 ECOG 1 ECOG 2 ECOG 3 ECOG 4	63 (31.7) 105 (52.8) 23 (11.6) 7 (3.5) 1 (0.5)
Charlson comorbidity index Median (IQR)	9 (8–10)
Primary tumour location Head or neck Body or tail Multiple Unknown	99 (49.7) 85 (42.7) 12 (6.0) 3 (1.5)
*De novo* metastases Yes No	131 (65.8) 68 (34.2)
Liver metastases Yes No	129 (64.8) 70 (35.2)
Number of sites of metastasis 1 2 or more	119 (59.8) 80 (40.2)
CA 19-9 (U/mL) Median (IQR)	540 (74.8–4,995.8)
Previous TEE Yes No	37 (18.6) 162 (81.4)
Khorana score Intermediate risk High risk Unknown	121 (60.8) 49 (24.6) 29 (14.6)
Baseline use of anti-thrombotic drugs Yes – Anti-platelet Yes – Anti-coagulant Yes – Both No	23 (11.6) 29 (14.6) 3 (1.5) 144 (72.4)

IQR: Interquartile range; TEE: Thromboembolic event

**Table 2.** Cumulative incidence of TEEs.

Time	Cumulative incidence – %	Cumulative incidence – 95%CI
1 month	10.1	6.4–14.8
3 months	16.5	11.7–22.1
6 months	19.3	14.0–25.3
12 months	24.3	18.4–30.6
24 months	30.2	23.5–37.0
36 months	31.8	25.0–38.9

**Table 3.** Characteristics of the TEEs.

Variable	*N* (%)
TEE (*N* = 199) Yes No	58 (29.1) 141 (70.9)
Type of TEE (*N* = 58)^#^ Venous Arterial Both	56 (96.6) 1 (1.7) 1 (1.7)
Site of TEE (*N* = 58)^$^ Lung Lower extremity Splanchnic vessel Subclavian/Jugular vein Heart Vena cava Aorta	21 (36.2) 17 (29.3) 12 (20.7) 6 (10.3) 3 (5.2) 2 (3.4) 2 (3.4)
Site of PE (*N* = 21) Central or segmental Subsegmental	19 (90.5) 2 (9.5)
Site of VT (*N* = 17) Deep Superficial	10 (58.8) 7 (41.2)
Symptoms at TEE diagnosis (*N* = 58) Yes No	22 (37.9) 36 (62.1)

# Venous TEEs include pulmonary embolism (mechanism is secondary to venous thrombosis)

$ Do not add up to 100% as some patients were diagnosed concurrently with more than one site of thrombosis TEE: Thromboembolic event; PE: Pulmonary embolism; VT: Venous thrombosis

**Table 4.** Outcomes and treatment characteristics of the TEEs.

Variable	*N* = 58 (%)
Outcome of TEE Outpatient management Inpatient management – no ICU Inpatient management – ICU Death Hospitalised – no repercussion Hospitalised – repercussion	23 (39.7) 7 (12.1) 2 (3.4) 3 (5.2) 21 (36.2) 2 (3.4)
Death due to TEE Yes No	3 (5.2) 55 (94.8)
Significant bleeding during anticoagulation^#^ Yes No	2 (3.4) 56 (96.6)
Treatment of TEE Rivaroxaban Warfarin Enoxaparin Apixaban Edoxaban Unknown	27 (46.6) 2 (3.4) 16 (27.6) 1 (1.7) 1 (1.7) 11 (19.0)
TEE relapse Yes – during anticoagulation Yes – without anticoagulation No	4 (6.9) 6 (10.3) 48 (82.8)

# Drop of 2 or more hemoglobin points secondary to bleeding

TEE: Thromboembolic event; ICU: Intensive care unit

**Table 5.** Time-dependent covariate Cox proportional hazard models for OS^#^ (*N* = 164).

	Univariate analyses		Multivariate analysis	
Variable	HR (95%CI)	*p*	HR (95%CI)	*p*
Age < 50 years 50–69 years ≥ 70 years	1.00 0.79 (0.43–1.45) 1.02 (0.55–1.88)	0.448 0.954		
Sex Male Female	1.00 0.85 (0.59–1.23)	0.399		
ECOG ECOG 0-1 ECOG 2-4	1.00 1.86 (1.16–2.97)	0.001	1.00 1.92 (1.19–3.10)	0.007
Liver metastases No Yes	1.00 2.18 (1.46–3.24)	< 0.001	1.00 1.85 (1.21–2.83)	0.004
Log_10_ (CA 19-9)	1.12 (1.05–1.20)	0.002	1.10 (1.02–1.18)	0.009
Number of sites of metastases 1 2 or more	1.00 1.74 (1.21–2.51)	0.003	1.00 1.43 (0.97–2.10)	0.069
TEE No Yes	1.00 1.88 (1.19–2.97)	0.007	1.00 1.59 (0.99–2.54)	0.051

# In this model, ECOG, liver metastasis, CA 19-9, number of metastatic sites and TEE were considered time-dependent covariates

ECOG: Eastern cooperative oncology group; TEE: Thromboembolic event

## Supplementary

**Supplementary Table 1.** Line of chemotherapy at the episode of TEE.

Line of chemotherapy	*N* (%)
Before first-line	17 (29.3)
During first-line	20 (34.5)
During second-line	15 (25.9)
During third-line	1 (1.7)
During fourth line	1 (1.7)
During best supportive care	4 (6.9)

**Supplementary Table 2.** Type of chemotherapy at the episode of TEE.

Type of chemotherapy	*N* (%)
FOLFIRINOX	11 (19.0)
Gemcitabine + Nab-Paclitaxel	10 (17.2)
FOLFOX	2 (3.4)
FOLFIRI	6 (10.3)
Gemcitabine	6 (10.3)
5-Fluorouracil	2 (3.4)
Before chemotherapy	17 (29.3)
During best supportive care	4 (6.9)

**Supplementary Table 3.** OR for TEEs with previous TEE.

	Univariate analysis		Multivariate analysis	
Variable	OR (95%CI)	*p*	OR (95%CI)	*p*
Age <50 years 50-69 years ≥ 70 years	1.00 2.51 (0.78–11.3) 3.28 (1.01–14.8)	0.163 0.073	1.00 1.13 (0.88–1.45) 1.17 (0.91–1.50)	0.338 0.223
Sex Male Female	1.00 1.23 (0.67–2.28)	0.504		
ECOG ECOG 0-1 ECOG 2-4	1.00 1.19 (0.50–2.66)	0.678		
Primary tumour site Head/uncinate Body/tail Multiple	1.00 1.80 (0.95–3.46) 2.36 (0.65–8.12)	0.073 0.174	1.00 1.07 (0.92–1.25) 1.19 (0.87–1.62)	0.374 0.277
Charlson Comorbidity Index	1.05 (0.81–1.34)	0.725		
Liver metastases No Yes	1.00 1.83 (0.95–3.70)	0.080	1.00 1.09 (0.94–1.27)	0.245
Log_10_ (CA 19-9)	1.42 (1.09–1.87)	0.010	1.03 (1.00–1.06)	0.030
Khorana score Intermediate risk High risk	1.00 1.62 (0.80–3.25)	0.175	1.00 1.13 (0.97–1.33)	0.124
Number of sites of metastases 1 2 or more	1.00 1.07 (0.57–1.99)	0.828		
Previous TEE No Yes	1.00 0.61 (0.29–1.32)	0.200	1.00 0.96 (0.80–1.48)	0.662

TEE: Thromboembolic event

**Supplementary Table 4.** Sub-distribution hazard ratios (SHR) for TEEs.

	Univariate analyses		Multivariate analysis	
Variable	SHR (95%CI)	*p*	SHR (95%CI)	*p*
Age < 50 years 50–69 years ≥ 70 years	1.00 2.06 (0.66–6.50) 2.74 (0.87–8.58)	0.220 0.084	1.00 1.73 (0.51–5.91) 1.94 (0.56–6.70)	0.380 0.290
Sex Male Female	1.00 1.13 (0.68–1.87)	0.650		
ECOG ECOG 0-1 ECOG 2-4	1.00 1.25 (0.65–2.42)	0.500		
Primary tumour site Head/uncinate Body/tail Multiple	1.00 1.75 (1.02–2.98) 2.24 (0.85–5.90)	0.041 0.100	1.00 1.33 (0.71–2.49) 2.36 (0.93–5.97)	0.380 0.071
Charlson Comorbidity Index	1.04 (0.87–1.25)	0.690		
Liver metastases No Yes	1.00 1.73 (0.97–3.10)	0.064	1.00 1.43 (0.78–2.65)	0.250
Log_10_(CA 19-9)	1.40 (1.10–1.78)	0.007	1.14 (1.02–1.27)	0.019
Khorana score Intermediate risk High risk	1.00 1.39 (0.81–2.40)	0.230	1.00 1.56 (0.85–2.87)	0.150
Number of sites of metastases 1 2 or more	1.00 1.06 (0.63–1.77)	0.820		
Previous TEE No Yes	1.00 0.61 (0.33–1.14)	0.120	1.00 0.82 (0.41–1.64)	0.580

TEE: Thromboembolic event

**Supplementary Table 5.** OS after the diagnosis of TEE according to line of therapy.

Line of therapy^#^	*N*	Median OS (months)	Median OS (95% CI)
Before first-line	17	9.1	0.6–13.4
During first-line	20	8.5	0.7–13.1
During second-line	15	1.1	0.5–2.7
During Best Supportive Care	4	0.3	0.1–NA
All patients	58	3.9	1.3–8.5

# We omitted the data for third and fourth line due to the low number of patients

CI: Confidence interval

**Supplementary Table 6.** Cox proportional hazard models for OS in landmark survival analysis.

	Univariate analyses		Multivariate analysis	
Variable	HR (95%CI)	*p*	HR (95%CI)	*p*
Age < 50 years 50-69 years ≥ 70 years	1.00 0.87 (0.48–1.58) 1.14 (0.61–2.08)	0.650 0.677		
Sex Male Female	1.00 0.91 (0.65–1.28)	0.597		
ECOG ECOG 0-1 ECOG 2-4	1.00 1.42 (0.85–2.38)	0.181	1.00 1.63 (0.97–2.76)	0.067
Liver metastases No Yes	1.00 2.02 (1.39–2.93)	< 0.001	1.00 1.67 (1.11–2.51)	0.014
Log_10_ (CA 19-9)	1.11 (1.04–1.19)	0.003	1.09 (1.02–1.17)	0.013
Number of sites of metastases 1 2 or more	1.00 1.45 (1.03–2.05)	0.034	1.00 1.35 (0.93–1.96)	0.117
TEE in 1^st^ month No Yes	1.00 1.30 (0.68–2.49)	0.422	1.00 1.23 (0.82–2.43)	0.560

TEE: Thromboembolic event
